# Complementary Assessment of Commercial Photoluminescent Pigments Printed on Cotton Fabric

**DOI:** 10.3390/polym11071216

**Published:** 2019-07-20

**Authors:** Selestina Gorgieva, Natalija Virant, Alenka Ojstršek

**Affiliations:** 1 Faculty of Mechanical Engineering, Institute of Engineering Materials and Design, University of Maribor, 2000 Maribor, Slovenia; 2 Faculty of Electrical Engineering and Computer Science, Institute of Automation, University of Maribor, 2000 Maribor, Slovenia

**Keywords:** photoluminescent pigments, cotton fabric, screen-printing, confocal microscopy, spectroscopy

## Abstract

The presented study focuses on photoluminescent pigments applied on cotton fabric by a screen-printed procedure using polydimethylsiloxane (PDMS) as a binder. Microscopic data depicts irregular shapes and relatively wide size distribution (3–80 µm) of pigments. Regarding composition, the Energy-Dispersive X-ray (EDX) and Fourier Transform Infrared (FTIR) spectroscopy data complement findings suggesting the presence of Eu-doped strontium aluminate in the yellow-green, calcium aluminate in the violet pigment, and metal oxides in the blue pigment. The optical properties of pigment-enriched PDMS-coated cotton fabric were assessed and reflectance intensity was found to be concentration-dependent only in the blue pigment. The luminescence decay data show that luminescence intensity decreased with the reduction of pigment concentration in the following order, yellow-green > blue > violet pigments. Relying on absorption and emission data of powdered pigments, the confocal microscopy enables visualization of the pigments’ distribution within a 3D image projection. This identifies the most homogeneous distribution in the case of the blue pigment, as well as the presence of a continuous fluorescing signal in the z projection when 5% pigment was used. This was, for the first time, presented as a powerful tool for non-destructive visualization of photoluminescent pigments’ spatial distribution when printed on textile (cotton) fabric. Finally, the photoluminescent PDMS coating demonstrates high washing and abrasion resistance, contributing to overall functionality of printed cotton fabrics when commercial types of pigments are applied.

## 1. Introduction

Pigments are a special type of water insoluble, organic and inorganic, colouring material [1], used for colouration of textiles, as well as metal, wood, stone, etc. Photoluminescent pigments are a subtype of textile pigments, and some could be used in creating very versatile, high-resolution “glow in the dark” patterns on textiles [2], as an alternative to light-emitting diodes (LEDs), electro-luminescent wires and optical fibres for designing light-emitting fabrics, being widely applicable as functional textiles [3], for decoration [4], military facilities, communication and transportation, fire emergency systems [5], etc. A relatively facile and convenient way of their immobilization onto textile fabrics, as far as simplicity of application is concerned, is by printing, and more than 80% of printed merchandise uses pigment printing, due to its versatility, and ease of near final print at the printing stage itself [6].

Photoluminescent pigments are synthetically-processed crystalline compounds, which absorb energy, followed by emission of light with lower energy and longer wavelengths [7]. Luminescence in solids arises generally when an inorganic host material is doped with a small amount of activator metal, which alters the electronic structure, resulting in a trap of charge carriers in metastable states upon excitation [8]. The presence of impurities and defects within pigments provides new states for electrons and holes, which could affect and change their motion, lifetime and energy transitions significantly [9]. Numerous host/activator combinations have been assessed for luminescence, although the development of luminescent materials requires a future effort in understanding of the relations between the host crystal structures and energy levels of dopant ions. Rare earth elements are among the most widely used activators within different hosts, generally, due to their high fluorescence efficiencies, when particles’ size is reduced to the nanosacale [10]. During the last two decades, a new generation of luminescent phosphorus, such as Eu^2+-^doped alkaline-earth aluminates, MAl_2_O_4_:Eu^2+^ (M ¼ Ca, Ba, Sr), have been studied and developed [11]. In contrast to the sulfide-based phosphorus, Ca, Ba and Sr-based aluminate phosphorus possess safer, chemically stable, very bright and long-afterglow photoluminescence, without any harmful radiation. As inorganic materials, they also overcome the issues related to organic pigments, such as poor photo- and thermal stability, gradual degradation, as well as poor washing and light colour fastness [11]. The specific (4f–4f) electronic transitions of rare earth ions enable its applicability in applications such as optical fibre amplifiers [12,13], state lasers [14,15], planar waveguides and compact microchip lasers [16], in vitro diagnostics [17], etc.

Beside chemical composition, the dimensions of photoluminescent pigments and their distribution within binders influence the print quality and final appearance of printed material greatly. However, techniques used for complementary assessment of pigments’ size and pattern distribution based on an insintric fluorescing property, when printed on fabric (i.e., in situ), are rather limited. Similarly, as in the investigation or artefacts, the aforementioned can be achieved by segmental, multistep and micro destructive sampling, followed by extensive laboratory analysis [18]. In art works and cultural heritage’ examination areas, several in situ identification techniques are in use (even limited to single point analysis), such as vibrational spectroscopy, laser-induced breakdown spectroscopy and X-ray spectroscopy for atomic-level analysis, as well as diffuse reflectance imaging, fluorescence and fluorescence lifetime imaging [18], etc. which are not employed at all for printed textiles’ evaluation. When only particles are considered, the Dynamic Light Scattering (DLS) methodology is applicable for nano pigments, preferably for monodisperse and, ideally, circular particles. The large scattering, even at very low concentrations (<<1%), is a limiting factor for the Ultraviolet–Visible (UV–Vis) spectrophotometry. Both Scanning Electron Microscopy (SEM) and Transmission Electron Microscopy (TEM) give distribution of patterns easily, however, the obtained information is limited to the examined surface, and they are not relevant for the luminescent effect examination. When commercial pigments with unknown composition and properties (particles’ size and their distribution, excitation wavelength, emission time, etc.) are employed, together with additives (binders, brighteners), the complexity of pigments’ evaluation increases exponentially. 

In the present work, a facile procedure for pigments’ screen-printing on cotton fabrics is presented, using three types of commercial luminescent pigments and polydimethylsiloxane (PDMS) as a binder. A comprehensive characterization of pigments and respective printed fabrics is carried out, using complementary microscopic (SEM and confocal microscopy) and spectroscopic (Energy-Dispersive X-ray (EDX), Fourier Transform Infrared (FTIR) and UV–Vis absorption, fluorescence and reflection spectroscopy) techniques. Special focus was given to the confocal microscopy, as a nondestructive tool for simultaneous examination of morphological and optical properties of photoluminescent pigments, and respective fabric prints in a 3D aspect. Confocal microscopy is already used for micro [19,20] autofluorescent pigments’ assessment [21], while assessment within textile fabrics has not yet been reported.

## 2. Experimental

### 2.1. Materials

We used 100% cotton fabric in plain weave, with a mass of 110 g/m^2^, warp density of 41 threads/cm and weft density of 38 threads/cm, and warp/weft fineness of 20 tex. Polydimethylsiloxane (PDMS), a two-part liquid elastomer kit, Sylgard 184 (DowCorning, Midland, MI, USA) that consists of a pre-polymer base and crosslinking curing agent in the ratio 10:1, was used as a binder for three commercially-available photoluminescent pigments of Sirius (Samson, Kamnik, Slovenia): violet, blue and yellow-green.

### 2.2. Fabrication of Luminescent Samples by Screen-Printing

Before a series of printing trials were started, the source fabric was washed at 40 °C for 30 min, using a solution of 2 g/L of standard neutral non-ionic washing agent, without optical bleacher, and, afterwards, rinsed in warm and cold water, and dried at ambient temperature.

Individual luminescent pigment in three concentrations: 1, 5 and 10 wt % (per weight of PDMS) was admixed in a PDMS binder for 5 min, using a high-speed paddle-stirring apparatus, to acquire homogenous pigments’ distribution. The prepared dispersions were applied onto cotton fabric, according to the flat screen-printing procedure, by means of a semiautomatic printing table, Johannes Zimmer, Austria. In order to achieve an even coverage over the entire sample, a PES 125, mesh was used, as well as pre-optimised parameters employed, i.e., roll-rod diameter of 15 mm, speed of 1 m/min, max magnet pressure, and 2 application layers. Additionally, printed samples were dried for 48 hours at ambient temperature, to form a sufficient cross-linked PDMS network.

### 2.3. Polydimethylsiloxane (PDMS) Coating

Determination of *mass per unit area* and *mass per unit length* were accomplished according to ISO 3801 standard, using Zweigle KG device (Zweigle Textilprüfmaschinen GmbH & Co KG, Reutlingen, Germany). Thickness of cotton fabric before and after PDMS coating was determined according to standard ISO 5084, using universal thickness gauge meter Luis Schoppen, Leipzig, Austria. Each sample was measured 5 times.

*Water contact angle (WCA)* measurement was performed using the sessile drop technique. An individual sample was placed on a horizontal table attached to a mechanical device on a Goniometer (DataPhysic Instruments GmbH, Filderstadt, Germany). A micro-drop with the volume of 0.3 μL MilliQ water was poured onto the fabric surface. The drop was illuminated by white diffuse light and observed with a tele-microscope. A clear image of the drop was transferred directly through a CCD-camera showing the drop profile. The contact angle was determined from the tangent to the drop at the three-phase contact line.

### 2.4. Scanning Electron Microscopy (SEM) and Energy Dispersive X-ray (EDX) Spectroscopy

Morphological analysis and elemental composition of selected photoluminescent pigments were accomplished by SEM coupled with EDX spectroscopy, using a Zeiss Gemini Supra 35 VP Scanning Electron Microscope (Carl Zeiss NTS GmbH, Oberkochen, Germany), equipped with an X-ray energy-dispersion spectrometer (EDX, Oxford Instruments NanoAnalysis & Asylum Research, model Inca 400, High Wycombe, UK).

### 2.5. Fourier Transform Infrared (FTIR) Spectroscopy

FTIR measurements of photoluminiscent pigments and printed textiles were accomplished using an FTIR System Spectrum GX spectrophotometer (Perkin Elmer, Waltham, MA, USA), with a Golden Gate ATR attachment and a diamond crystal. The absorbance spectra were obtained within the range of 4000–650 cm^−1^, with 32 scans and a resolution of 4 cm^−1^.

### 2.6. Confocal Fluorescent Microscopy (CFM)

Cotton fabrics printed with three different photoluminescent pigments in all three concentrations were assessed by Confocal Fluorescent Microscopy (CFM). Square (1 cm × 1 cm) fabric pieces were positioned on a transparent glass holder above the 20 × (dry) objective of an inverted CFM Leica TCS SP5 II (Leica Microsystems, Wetzlar, Germany), equipped with an LAS AF software program. The photoluminescent pigments were excited with an argon laser (λ_ex_ = 458 nm), while the obtained signal was detected by a hybrid HyD3 detector, with a pre-set emission range from 500 to 550 nm. High-resolution images (1024 × 1024 pixels) were obtained by image-adjusted light gain and 8 × line averaging. Each sample was depicted at several positions, in order to check the printing uniformity in the x–y direction, while in the z-direction (sample thickness), the confocal mode with 10 µm stack thickness was applied, and assessed additionally by the depth (colour) codding function. By moving the focal plane in the z direction, up to 30 optical slices were obtained, and combined further in a 3D image stack for digital processing. The bright field images were captured in parallel, to depict also the pigment-lean areas of printed cotton fabrics, using a Dodt detector. 

The images from both the fluorescence and bright field channels were used in split or overlaid mode for further assessment by the ImageJ program, an image processing program, designed for scientific multidimensional images, in particular, the z-project function (for tracking of pigments` distribution within the cotton fabric). 

In parallel to the fabrics, the pigments–PDMS binder dispersion was also coated onto thin glass (only at a concentration of 1 wt %). The particle analysis plug-in, also available within the ImageJ program, was employed for the assessment of size and distribution of pigments within the PDMS binder on the glass. Pure cotton fabric and PDMS were analysed as controls.

### 2.7. Optical Properties

#### 2.7.1. Ultraviolet–Visible (UV–Vis) Spectroscopy: Absorbance and Fluorescence Intensity Measurement

UV–Vis absorbance intensity of photoluminiscent pigments and printed cotton fabrics was measured on a spectrophotometer, Infinite M200 Pro (Tecan Trading AG, Männedorf, Switzerland), equipped with a plate reader. All samples were scanned within wavelengths of 230–800 nm. The emission intensity (for the fluorescence data) was recorded under different excitation wavelengths (the estimated λ_max_ at absorbance measurement), using a black wall microplate (Nunc 96, flat), and the same equipment.

#### 2.7.2. Reflectance

The reflectance profile of the printed cotton samples in the 200–700 nm wavelengths was recorded on a Lambda 900 UV–Vis Near-InfraRed (NIR) spectrophotometer (Perkin Elmer, Waltham, MA, USA), equipped with an integrated sphere, at a scanning speed of 450 nm per min, and a resolution of 10 nm.

#### 2.7.3. Afterglow Decay Kinetics

A qualitative assessment of luminescence decay of cotton samples, printed with a combination of PDMS and individual photoluminescence pigment in three concentrations, was performed by initial, 5 min exposure of samples under UV light with a wavelength of 366 nm, within a UV chamber. Afterwards, samples were exposed immediately to deep dark within a chamber, and after 20 seconds and 1 min, the photos were taken. For relative quantification of time-dependent luminescence decay, same samples were also evaluated on a Tecan Infinite M200 Pro microplate reader (Tecan Trading AG, Männedorf, Switzerland) in luminescence mode, without using emission filters. Printed cotton fabrics were again excited under the same conditions as for the qualitative assessment, and loaded immediately into the instrument, to commence measurement. Each decay curve was normalized by its initial intensity. Before afterglow decay measurements, all printed samples were exposed to darkness for a two-day period, in order to attenuate any afterglow illumination completely.

### 2.8. PDMS Coating Stability

For coating stability on washing, the PDMS coated cotton fabrics were washed according to standard ISO 105-C06, up to 30 times, at a temperature of 40 °C for 30 min, in a Labomat (W. Mathis AG, Oberhasli, Switzerland), using a solution of 1 g/L of standard reference detergent without optical brighteners, and a liquor-to-fabric weight ratio of 50:1. After each washing cycle, the samples were rinsed several times in a tap water, and dried at room temperature. Mass loss and WCA determination of the samples were accomplished before and after several set of washing cycles (up to 30^th^).

The abrasion resistance of coatings was evaluated by the Martindale method, according to standard EN ISO 12947-3, using 1.000, 5.000, 10.000 and 20.000 rubbing cycles. The mass loss of the samples was determined after each set of rubbing cycles.

## 3. Results and Discussion

### 3.1. Characterization of Photoluminescent Pigments

**Figure 1:**
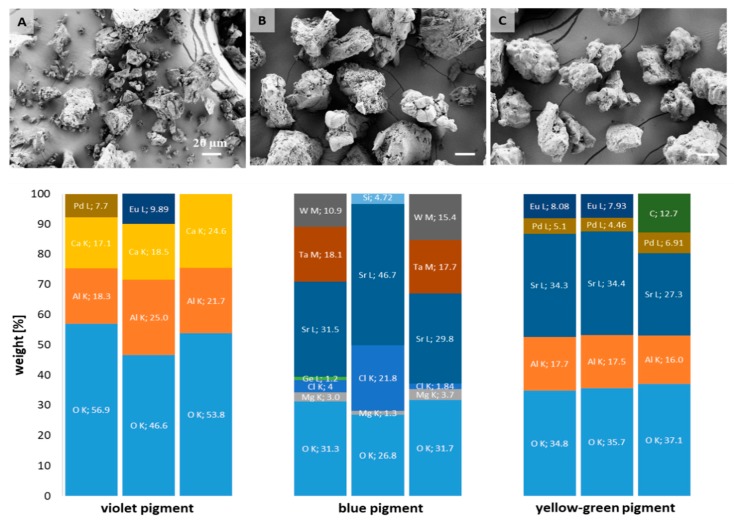
Scanning electron microscope (SEM) images of violet (**A**), blue (**B**) and yellow-green pigment (**C**) at 1000× magnification, with elemental (EDX) analysis (wt %) extracted from 3 images of each pigment, presented below the respective pigment. The scale bar corresponds to 20 µm in each micrograph.

The morphology of three commercial, powder-like photoluminescent pigments and their atomic composition were assessed initially by SEM, coupled with EDX spectroscopy. The micrographs obtained in Figure 1 (above) reveal irregular shape, as well as high heterogenicity in all pigments, which is generally associated with their conventional production procedure, including solid-state bulk synthesis, and high temperature calcination. Violet pigment particles, in particular, demonstrated the widest size distributions and presence of particles with a size of 1–100 µm, being above the reported dimensions for commercial phosphorescent pigments [8]. In the case of the other two pigments, visually distinct segments can be observed within the same particle; from fine powders to globules, and even needle like crystals (blue pigment, B). The size and shape of pigments were identified as factors influencing light absorption and emission intensity; however, a major difference was expected from the presence of particles below 0.5 µm [22], which, according to microscopy data, do not dominate in any of the pigments used.

The EDX elemental distribution analysis were carried out with the intention to identify the presence of particular elements within commercial powder compounds, to which the luminescence effect discussed later will be assigned, rather than to identify their compositions fully, being out of this paper’s scope. Indeed, luminescent properties of pigments depend strongly on the chemical composition of the host material, as well as on the presence of particular dopant materials [23]. Due to the depicted heterogenicity, three regions of interest were selected for elemental analysis, including several pigment particles rather than particle sections, which gives a broader and more general view about the distribution of detected elements. Column charts (Figure 1, below) reveal relative (weight) percentage of elements within each type of pigment. The presence of the rare earth element europium (Eu), known as a photoluminescent pigments’ dopant [24], was identified in the yellow-green and violet pigments. This element has strong luminescence intensity, which is affected by its oxidation state (the Eu^2+^ wavelength position is matrix-sensitive, while the Eu^3+^ emission position is not affected by change in the matrix [23]), as well as concentration. Besides Eu, the yellow-green pigment contains strontium (Sr), aluminium (Al) and oxygen (O), which suggest the presence of strontium aluminates. They are well-known metal aluminates, with phosphorescent properties, long afterglow at room temperature, and a broad spectrum distribution of luminescence from the blue to green region, and emission maximum, which, generally, depends on the host [25,26]. The increased molar ratio of SrO to Al_2_O_3_ in synthetic process results in strontium aluminates with numerous stoichiometries: SrAl_12_O_19_, SrAl_4_O_7_, Sr_4_Al_14_O_25_, SrAl_2_O_4_ and Sr_3_Al_2_O_6_ [27]. Even EDX provides a rough estimation of atomic percentages; the EDX data suggest the presence of an SrAl_4_O_7_ structure as dominant, although, the presence of other types is also not excluded. In general, the aluminates are recognised as low cost, stable, anti-radiation and eco-friendly hosts of rare earth luminescent materials, being synthetised mainly as mixed phase compounds [24]. In the case of the violet pigment, the calcium aluminate elements (Ca, Al and O) were identified, presumably again, as mixed (CaAl_2_O_4_, CaAl_4_O_7_, CaAl_12_O_19_, Ca_3_Al_2_O_6_, Ca_12_Al_14_O_13_), rather than a particular phase. In this pigment, the Eu presence was spotted in 1 of 3 regions, with an even higher percentage compared to the yellow-green pigment. Considering the blue pigment, where Al is absent, the aluminates’ presence is excluded, although the presence of different metals and O imply the possible presence of metal oxides (MgO, SrO), as luminous materials [28], with an even more complex luminous behaviour. 

**Figure 2:**
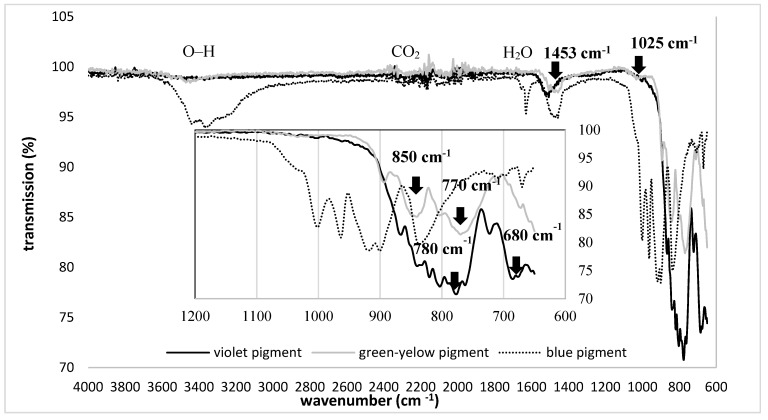
Fourier Transform Infrared (FTIR) spectral lines of violet, blue and yellow-green luminescent pigment powders.

The molecular composition of pigments was assessed further by FTIR spectroscopy in transmission mode, and the results were presented in Figure 2. Common bands were identified in all pigments, at ~1630 cm^−1^ attributed to water presence 1340–1572 cm^−1^ (symmetric and asymmetric stretching vibration of CO_2_) and ~3460 cm^−1^ (O–H bond vibration from surface-adsorbed water). In the range of 1000 to 600 cm^−1^, several absorption bands were observed, related to the stretching vibration of metal oxygen bonds (Al–O, Sr–O and Sr–O–Al) [29]. 

In the yellow-green pigment, the bands at 850 and 770 cm^−1^ are assigned to the stretching vibration modes of SrAl_4_O_7_ [30], which confirmed the EDX findings (Figure 1). In addition, bands at 1025 and 1453 cm^−1^ within the same pigment, are related to the Al–O stretching vibration from the strontium aluminate. Both vibrations, with slight shifting, were detected in the IR spectral lines of violet pigment, corresponding to Al-O vibration within the calcium aluminates, also demonstrated further by bands at 680 and 780 cm^−1^. In the case of the blue pigment, the multiple bands stated above were noted in the metal-oxide range, which is a contribution of both oxides as such, or oxides potentially-doped with heavy alkaline-earth metals (such as Sr), used for luminescence enhancement [31]. The presence of functional groups on pigments’ surfaces can affect luminescence properties, as they are considered as special defects. Indeed, they are relatively light compared to rare earth ions, and, as such, have high vibration energy. The luminescent centre near the surface couples with the vibration modes of those functional groups. The photon energy provided by these vibration modes is relatively strong, so the electrons of the luminescence centre can relax easily to the lower energy level in non-radiative relaxation form, and will not give out light due to the fluorescence quenching [32].

**Figure 3:**
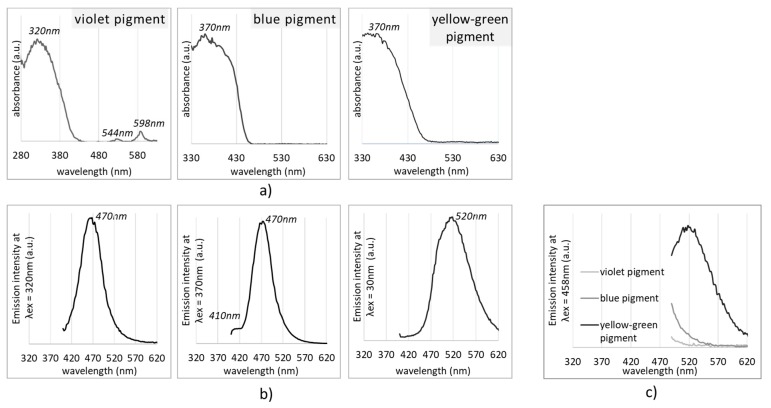
Absorption (**a**) and emission spectra at λ_ex_ = 370 nm (**b**) and λ_ex_ = 458 nm (**c**) of violet, blue and yellow green pigments. Spectral lines are normalised at maximum intensity.

Prior to analysis on fabrics itself, the pigments’ spectral profiles (absorption and emission) were assessed to inform further studies on printed cotton fabric. According to Figure 3a, the main absorption peak at 320 nm was detected for the violet pigment, and at 370 nm for the blue and yellow-green pigments, which corresponds with exciting data for strontium aluminates absorption [33] present in yellow-green pigment. Exciting the pigments at corresponding λ_max,_ the emission spectral distributions were obtained (Figure 3, with emission maxima at 470 nm and 520 nm for the violet and yellow-green pigments, respectively), corresponding to the ground up to excited state transition of luminescent centres. The prominent peak at 520 nm was attributed to the transition of the Eu luminescent rare earth element [34]. 

**Figure 4:**
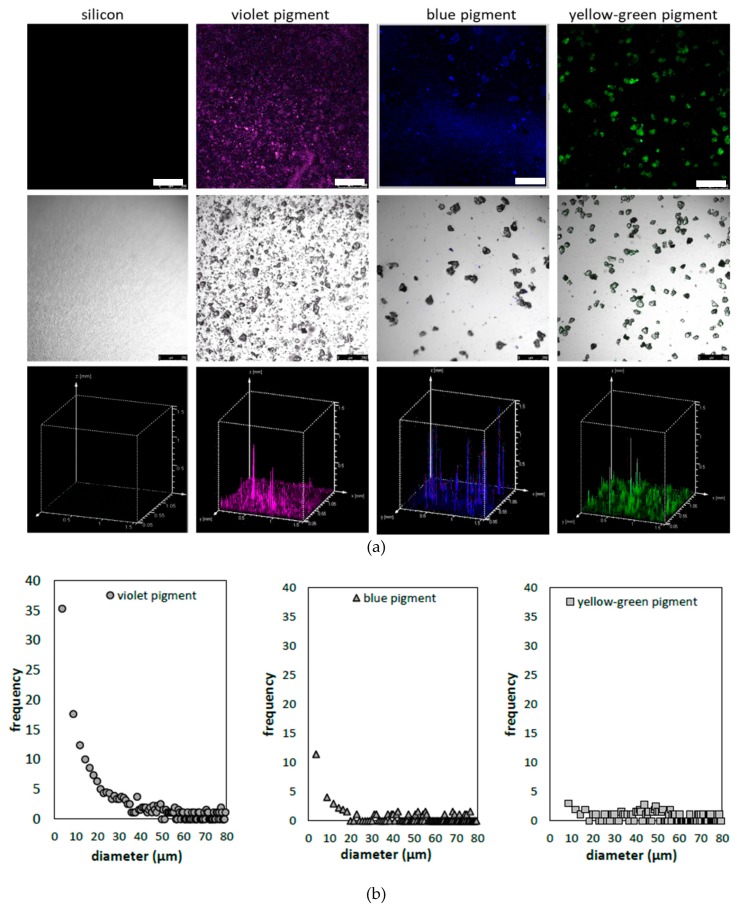
(**a**) Confocal microscopy micrographs of luminescent pigments dispersed within the polydimethylsiloxane (PDMS) binder at 5 wt % concentration. Top: fluorescence channel; centre: fluorescence channel combined with bright field images and bottom topological 3D view, displaying two-dimensional height profiles using the fluorescence intensity as the third dimension. (**b**) Bar diagram for particle size distributions in the violet, blue and yellow green pigments at 5 wt % concentration within the PDMS binder. Data are averaged from 5 micrographs with dimensions 1.5 mm × 1.5 mm. Scale bars correspond to 250 µm.

In addition, even if not optimal, we inspected the emission signals in the range of 500–600 nm under the same (458 nm) excitation for all pigments (Figure 3c), with the aim to find out if they were sufficient for further confocal microscopy imaging, using argon laser excitation. Such laser was used to avoid the autofluorescence of cotton (or its finishing additives), being relevant in post-printing analysis. Confocal microscopy was used to assess the pigments’ size distributions when dispersed within the PDMS binder, which was used in the following textile-printing process. This method utilises the emission signal from the luminescent particles, when excited at particular wavelength, offering complementary assessment of both distribution of particles in the horizontal (xy) position, and emission intensity presented as the z dimension within the topographic profiles (Figure 4a, bottom). 

The PDMS binder in the control (pigment-free) sample obviously did not generate any background emission, while the clear signal was obtained for each pigment at a selected broadband detection (500–550 nm). The micrographs obtained from the merged (fluorescence and bright field) channels were used further for the calculation of pigments’ size (in µm), as well as diameter calculated from the area, considering circular particles, using the ImageJ plugin Analysis Particles. The results identify wide size distribution within all pigments, of 3–80 µm in range. This corresponds to the size reported for Eu-doped strontium aluminates, where particle size is within the 20–100 µm range [35]. In the yellow-green pigment, the smallest fraction (d ~3µm) is missing, the same being dominant in the other two pigments. The size distribution of the violet pigment differs significantly compared to the other two pigments, in terms of count, being >3× higher for particles in the 3–30 µm range. Moreover, the 3 µm fraction dominates greatly within the pigment particles, which may interfere with the emission intensity [36]. 

If compared to the SEM microscopy, where data collection requires more effort, the confocal microscopy provided a large data set with satisfied resolution, where dominant smaller particles can be resolved even at 10× magnification, excluding the sample preparation step. As such, they will also be applied for their identification within the cotton print, being, for the first time presented in this area.

### 3.2. Characterization of Luminescent Pigment Printed on Cotton Fabrics

PDMS coating under optimised screen-printing conditions (described within experimental section) increase the thickness of cotton fabric for ~10.5%, in same time reducing the hydrophilicity as detected by WCA increase from 0 to 135.3 ± 3.8 °C. Presence of photoluminescent pigment did not significantly deviate from this value, due to their low weight, as well as complete inclusion within the coating.

**Figure 5:**
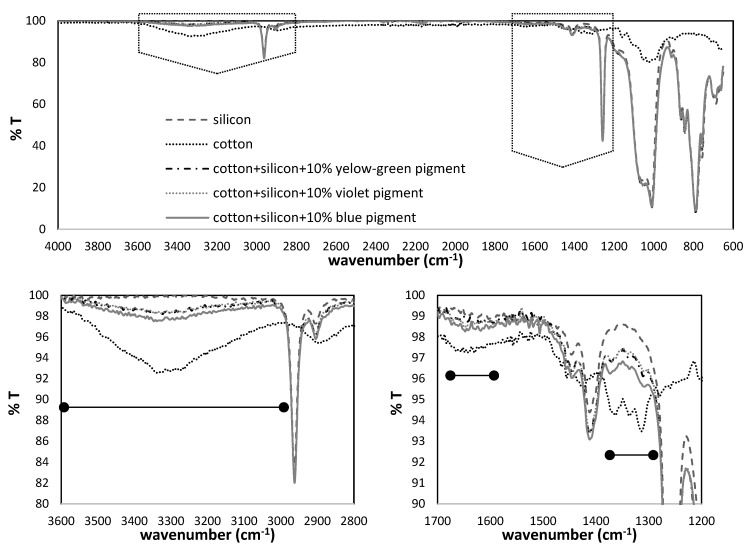
FTIR spectra of cotton fabric, before and after screen-printing, together with the PDMS binder, containing 10 wt % of blue, violet and yellow-green luminescent pigments. The PDMS binder is presented as the control. Regions of interest are presented with a higher resolution.

The presence of the PDMS binder, as well as photoluminescence pigments on cotton fabric, was directly evaluated by FTIR as presented on Figure 5. The spectrum of the control cotton fabric displays several characteristic bands related to the cellulose structure: O–H stretching (broadband in 3500–3000 cm^−1^ region) and bending (1640 cm^−1^) vibrations of physically adsorbed water, C–H stretching (3000–2800 cm^−1^), symmetric C-H bending and C–C/C–O skeletal vibrations (at 1425 and 1315 cm^−1^, respectively), C–O–C skeletal vibration (1103 and 1160 cm^−1^), asymmetric in-plane ring stretching (1053 cm^−1^), C−O stretching (1030 cm^−1^), and asymmetric out-of-phase ring stretching vibration (895.8 cm^−1^). The presence of the PDMS binder is demonstrated by the bands related to C–H stretching (2962 cm^−1^), Si–CH=CH_2_ (1410.9 cm^−1^), asymmetric CH_3_ stretching in the Si–CH_3_ bond (1258cm^−1^), Si–O–Si stretching (~1050 and 1011 cm^−1^), CH_3_ rocking and Si–CH_3_ stretching vibration (790 cm^−1^) [37]. 

From Figure 5, it can be perceived that further enlargement of pigments concentration up to 10 wt % had not affected the ultimate molecular fingerprint significantly, which implied the complete inclusion of particles within the PDMS bulk. In addition, the cotton fingerprint vibrations in the 1300–1400 cm^−1^ region are still visible in the PDMS-printed fabrics (red line), retaining partial hydrophilicity of the printed fabric, being evidenced by the presence of water-related vibrations (indicated by the blue line).

**Figure 6:**
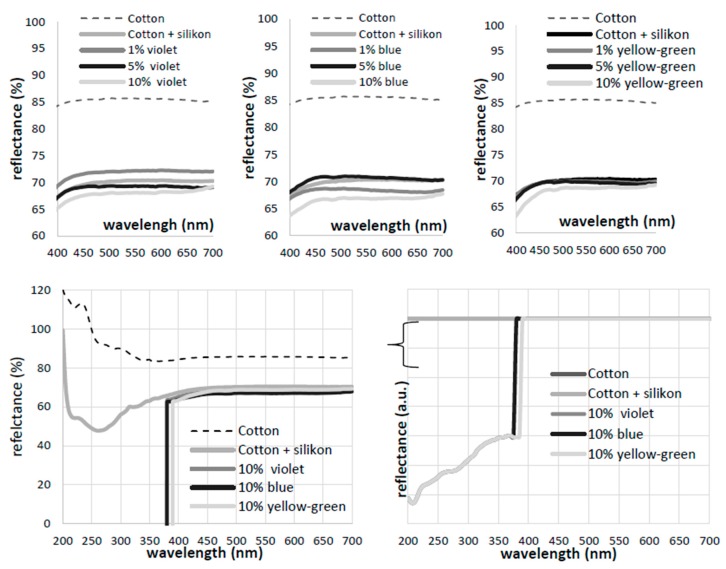
Reflectance spectra of cotton fabrics before and after screen-printing with PDMS containing 0, 1, 5 and 10 wt % of violet, blue and green-yellow photoluminescent pigments.

The reflectance spectrum was obtained for the cotton control, as well as for the PDMS-printed and luminescent pigment-printed fabrics (Figure 6). The absence of a local reflectance maximum at a wavelength of ~440 nm, as most often related to the optical brightener [38], exclude the potential presence of an optical bleaching agent, which could, if present, interfere with the photoluminous effects of the pigments. Moreover, the presence of the PDMS binder reduced the reflectance of cotton fabric in the whole measurement range (with a peak at ~260 nm), appearing visually as “more transparent”, i.e., less reflective than the native fabric. PDMS addition increased the flatness of the cotton fabric, reducing the surface roughness on the printing side, and also covering the surface dangling fibres, which also supplement the scattering.

Reflectance curves in a visible range (400–700 nm) indicate the reduction of reflectance from the non-planar cotton surface by PDMS printing. Employment of three pigments in concentrations of 1, 5 or 10 wt % reduced the reflectance in a concentration-dependent manner; being less pronounced in the yellow-green pigment. 

**Figure 7:**
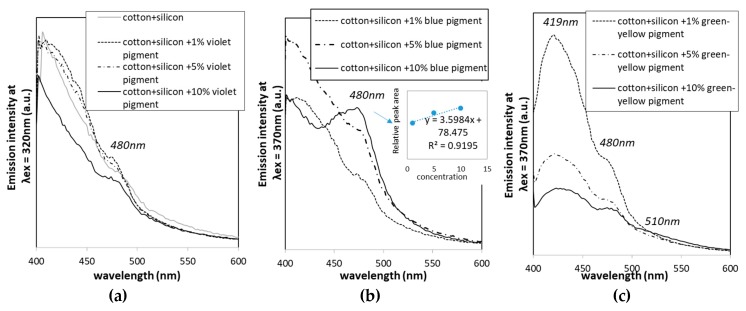
Emission spectra under pre-selected excitation wavelengths at 320 and 370 nm, obtained for the cotton printed with PDMS and different concentrations (1%, 5% and 10%) of blue (**a**), violet (**b**) and yellow-green luminescent pigments (**c**).

The emission spectra were also evaluated for the printed cotton samples (Figure 7), similarly as for pure pigments (Figure 3b,c), using the same λ_ex_ values, and PDMS-printed cotton as a control. The 10 nm blue shift (from 470 nm down to 480 nm) of pigment-printed cotton in comparison to the violet and blue pigments, and 10 nm red shift (from 520 nm up to 510 nm) using the green-yellow pigment, are presumably related to the incorporation of pigments within the PDMS binder. An increase of pigment concentration from 1 wt % up to 10 wt % demonstrated linear relation, with an increase of emission intensity only in the blue pigment, considering the major peak at 480 nm, which was not perceived in the case of the other two pigments, where just small, and not concentration-dependent, changes were observed.

**Figure 8:**
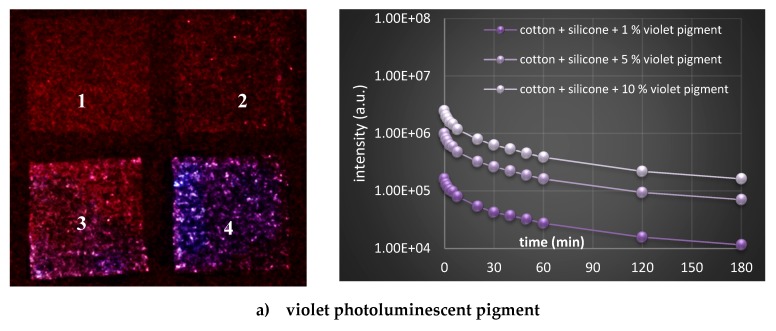

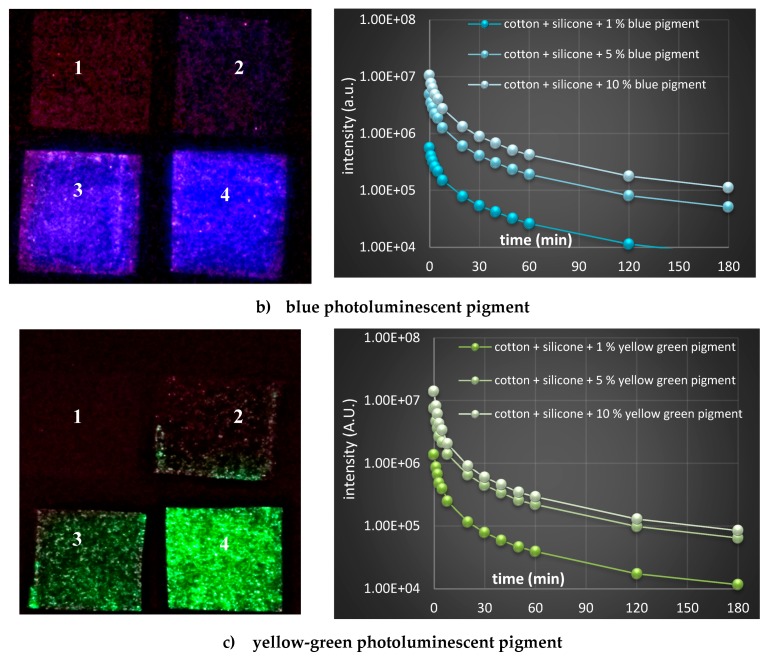
(left) Photographs depicturing differentially printed cotton fabrics, excited within a UV chamber (366 nm) for 5 min. Cotton printed with PDMS (1) 1 wt % (2), 5 wt % (3) and 10 wt % of violet (**a**), blue (**b**) and yellow-green photoluminescent pigment (**c**). (right) Luminescence decay curves were measured for the same samples in a 180 min period (after 1, 2, 3, 5, 8, 20, 30, 40, 50 60, 120 and 180 min). Luminescence curves for the cotton and cotton printed with PDMS without photoluminescent pigments are below the threshold value, i.e., luminescence of the microplate itself.

Luminescence decay curves were obtained from the relatively wide time span measurement- from 1 up to 180 min, and presented in Figure 8 (right column). For the visualization of decay in 20 s, the photographic images were collected (Figure 8, left side), and exponential luminescence decay was observed in all cases, irrespective of pigment types and concentrations. Data show an expected luminescence intensity decrease (at time point 1), with a reduction of the pigment’s concentration (irrespective of pigment type), while, among different pigments, the intensity decreased in the order yellow-green > blue > violet at the same concentrations. The presence of rare earth aluminates activated by Eu brought the highest emission intensity in the yellow-green pigment, as well as the longest afterglow [33]. At very low concentration of dopant ions (e.g. rare earth elements), the fluorescence decay curves can be fitted to a single exponential function, which indicates that energy transfer between luminescent ions is not dominant, which could also be seen in our study [16].

**Figure 9:**
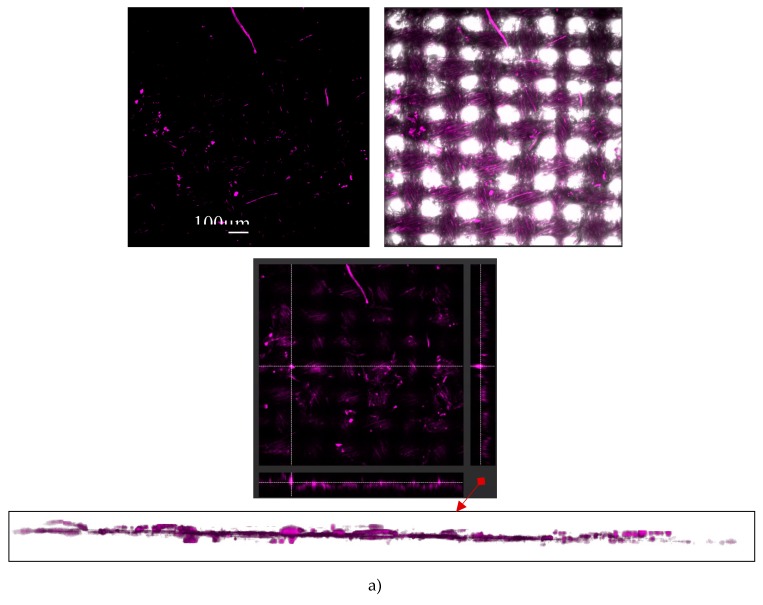

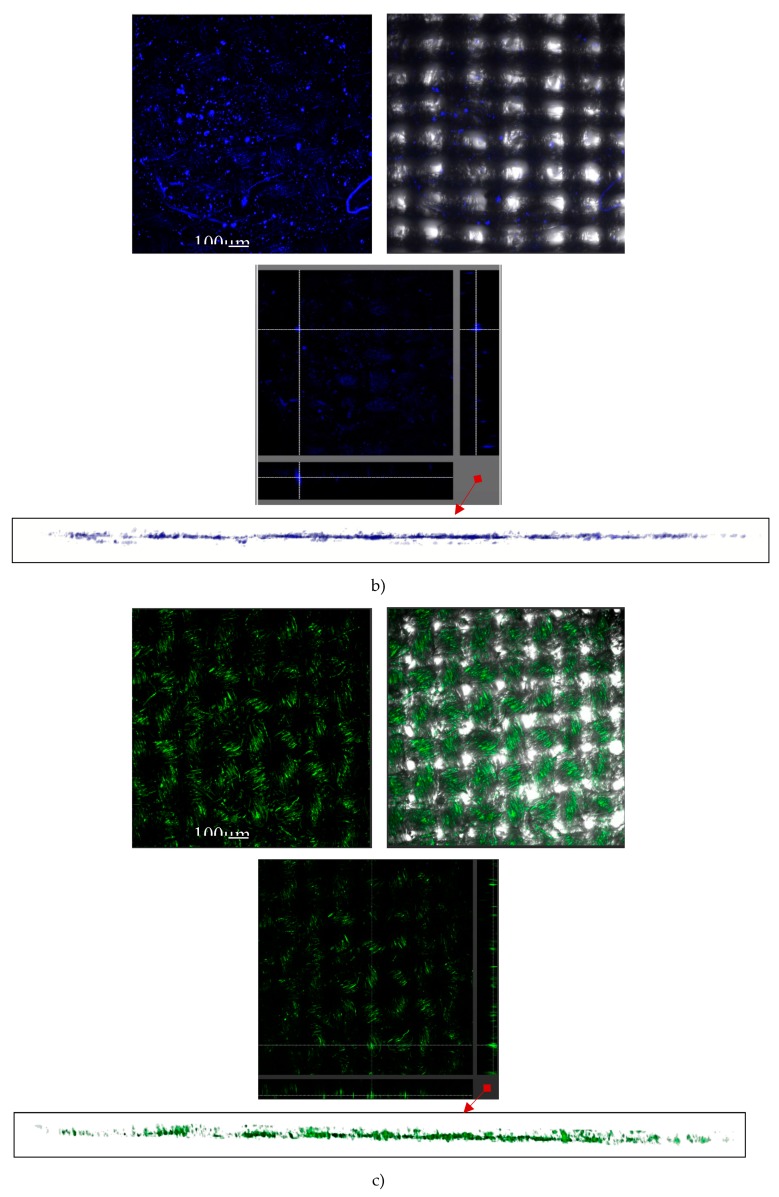
Confocal microscopy images: maximum projections (left) with combined, brightfield and fluorescence channel (middle) and orthogonal projections (right) of luminescent violet (**a**), blue (**b**) and yellow-green (**c**) pigments, printed on cotton. The bottom inserts are the xz view from a 3D volume viewer obtained by image analysis.

Confocal microscopy was used for the investigation of the distribution of pigments in both the horizontal (xy) and vertical (xz/yz) directions of the cotton fabric, and the results were presented in Figure 9. Confocal microscopy permits depiction and localization of the fluorescing compounds unambiguously at light microscopy resolution [19], offering a non-destructive method for in-depth imaging, without demanding preparation steps. This is due to the point-by-point illumination of samples, and rejection of out of focus light, which enables the 3D imaging [39]. The 3D image is built up by sequential recording of fluorescence emission from a large number of points in a sample in all three dimensions, being achieved by controlled movement of the focus point of the laser beam by mirrors, and by moving the sample in the third dimension by automated stage [40]. The same imaging parameters were applied for both pigments and printed cotton fabrics. Under specified excitation conditions (λ_ex_ = 458 nm), the particles can be visualised clearly and distinguished from the cotton fabric, which itself exhibited a very low background signal. The increase in pigment concentration from 1 up to 10 wt % was not supplemented by a fluorescence signal to a particular level (Supplementary data 1), as could also be observed in the fluorescence emission measurement. In general, the recipes for the printing of photoluminescent pigments on textile fabrics recommend suboptimal concentration of pigment, e.g. 30 wt %, which is higher than in our study, but increases the cost of the procedure, and, at the same time, does not essentially improve the optical effect, which we evidenced here. Figure 9 depicts the maximum intensity from the consecutive images, comprising the z stack (left) and orthogonal projection (right) from a selected image within the same stack. Relatively homogeneous distribution of pigments was observed from the maximum intensity image, as within single pigment analysis, which points out the suitability of the selected pigment-binder-printing procedure. Pigments were observed within an inspected thickness (<300 um), and not through the whole fabric thickness, which is in the function of an objective aperture. This conclusion was confirmed further by the application of deep colour-coding, where different colours correspond to the fluorescence signal from different z (thickness) positions (Supplementary data 2). In the case of the violet pigment, the most heterogenic distribution was seen in the xy plane compared to the other two pigments. The orthogonal projections, as well as fully depicted (red arrow) thickness view, imply the presence of a continuous fluorescing signal in all cases at pigment concentration of only 5%. The blue pigment was the most homogeneously distributed, and as in the violet pigment print, the presence of fully fluorescing fibres was observed, most probably on account of the finest pigment particles covering the fibres dangling out of the fabric. 

Photoluminescence-enabled PDMS coating on cotton fabric withstand up to 30 washing cycles without significant mass loss, while keeping same level of hydrophobicity (WCA ~constant 131°), and in the same time significantly improving the abrasion resistance. Indeed, after 20,000 rubbing cycles, the mass loss of PDMS-coated cotton fabric was 34% lower than non-treated cotton fabric (having ~ 10% weight loss after mentioned rubbing cycles). This are attributes arising from PDMS, rather than pigments itself, yet contribute to overall functionality of printed fabrics.

## 4. Conclusions

Three commercial photoluminescent pigments were screen-printed on to cotton fabric and comprehensively evaluated, using several advanced microscopic and spectroscopic techniques, in powdered form, as well as when applied onto the cotton textile fabric. Confocal microscopy was particularly emphasised, and used for the first time in the evaluation of pigments’ size and distribution, and for the simultaneous visualization of their emission intensity. The non-destructive assessment of spatial 3D distribution of pigments within cotton textile fabric is also not reported as an application of confocal microscopy, and as such, in combination with complementary analytical set-up can be applied further in analysis related not only on textile fabrics, but even to more specific areas related to textile print, artefacts conservation and similar. Related to the printing procedure itself, this study demonstrates an effective and facile procedure involving commercial types of pigment for processing durable and luminescent- effective textile prints.

## Supplementary Materials

The supplementary materials are available online at https://www.mdpi.com/2073-4360/11/7/1216/s1.
