# Supplementary material for: Complementary Assessment of Commercial Photoluminescent Pigments Printed on Cotton Fabric
Source: Polymers (Basel). 2019 Jul 20;11(7):1216. doi: 10.3390/polym11071216 (PMC6680420; doi:10.3390/polym11071216)
Supplement: Supplementary file 1 [file polymers-11-01216-s001.pdf]

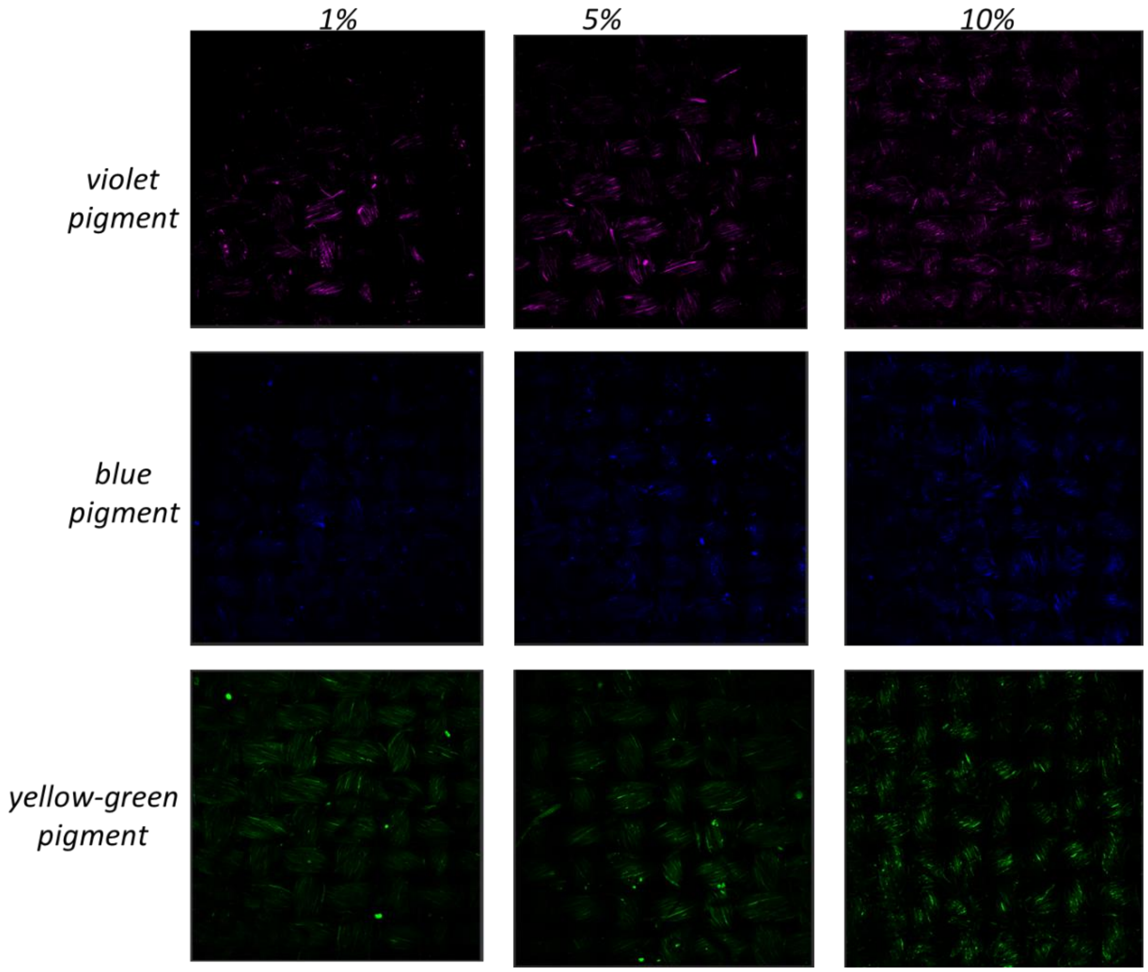

Supplementary data 1. Confocal microscopy images for 1%, 5% and 10% of violet, blue and yellow-green pigments printed on cotton fabric by screen printing technology, using PDMS binder.

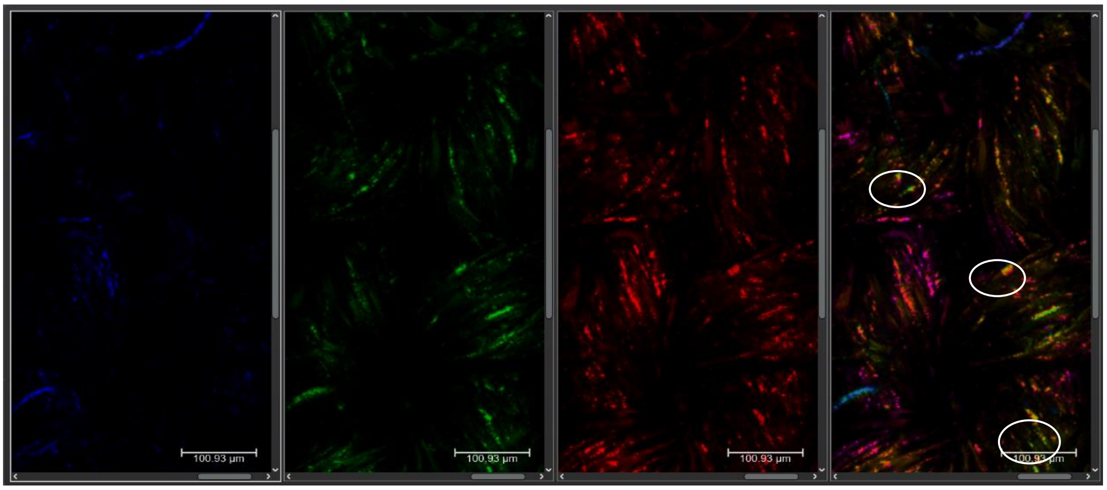

Supplementary data 1. Deep color-coded, zoomed images obtained from confocal microscopy, visualizing presence of yellow-green pigment within cotton fabric, when screen printed at 10% concentration.
